# What are the patient-reported outcomes, functional limitations, and complications after lesser tuberosity fractures? a systematic review of 172 patients

**DOI:** 10.1016/j.jseint.2021.02.016

**Published:** 2021-04-20

**Authors:** Reinier W.A. Spek, Bram J.A. Schoolmeesters, Chantal den Haan, Ruurd L. Jaarsma, Job N. Doornberg, Michel P.J. van den Bekerom

**Affiliations:** a Medical Doctor, Department of Orthopaedic Surgery, Flinders Medical Centre, Adelaide, Australia; b Clinical Librarian, OLVG, Amsterdam, The Netherlands; c Orthopaedic Trauma Surgeon, Department of Orthopaedic Surgery, Flinders Medical Centre, Adelaide, Australia; d Orthopaedic Surgeon, Shoulder and Elbow Expertise Centre, Department of Orthopaedic Surgery, OLVG, Amsterdam, The Netherlands

**Keywords:** Lesser tuberosity fracture, Systematic review, Subscapularis avulsion, Outcomes, Adults, Posterior shoulder dislocation, Pediatric patients

## Abstract

**Background:**

Lesser tuberosity fractures are relatively rare, with an incidence of 0.46 per 100,000 persons per year. This systematic review was performed to address patient-reported outcomes (PROMs), shoulder function, and complications after lesser tuberosity fractures in pediatric and adult patients, as well as patients with an associated posterior shoulder dislocation. Within these groups, identical outcomes were evaluated for nonoperative, surgical, acute and delayed treatment.

**Method:**

A comprehensive search was carried out in multiple databases. Articles were included if patients sustained a lesser tuberosity fracture without a concomitant proximal humerus fracture. There were no restrictions on age, type of treatment, fragment displacement, time to presentation, or associated injuries.

**Results:**

One thousand six hundred forty-four records were screened for eligibility of which 71 studies were included (n = 172). Surgical treatment was provided to 50 of 62 (81%) pediatric patients, 49 of 66 (74%) adults, and 34 of 44 (77%) patients with an associated posterior shoulder dislocation. In the pediatric group, the mean of PROMs was 94 (range 70-100) and among adults 89 (range 85-100). In the posterior shoulder dislocation group, 89% did not regain full range of motion and the complication rate was 17%. In pediatric patients, surgery was associated with fewer complications (*P* = .021) compared to nonoperative treatment.

**Conclusion:**

Pediatric patients have excellent outcomes after lesser tuberosity fractures and respond well to surgical treatment. Adults have acceptable outcomes but patients with an associated posterior shoulder dislocation have impaired range of shoulder movement and are more likely to develop complications.

The lesser tuberosity (LT) is a bony prominence on the proximal humerus, and important for stability and shoulder internal rotation as it accommodates insertion of the subscapularis tendon. Therefore, a fractured LT may cause shoulder dislocation or restricted internal rotation due to subscapularis insufficiency. LT fractures may occur in the setting of acute trauma—typically with the arm in 90° abduction and external rotation—or indirect, after repetitive stress caused by excessive overhead use of the arm such as in athletes of throwing sports or adolescents.^21^ LT fractures are rarely seen in clinical practice, and likely to be missed as they are hard to detect on radiographs.^24^^,^^65^^,^^82^ Moreover, missed or inadequately treated LT fractures may cause disabilities such as pain, muscle weakness, and impaired shoulder movement due to the development of bony exostosis which has been described up to 20 years after the initial trauma.^19^

Patients can be treated nonoperatively, arthroscopically with suture anchors or via open reduction with internal screw fixation, tension band stabilization, or transosseous sutures. A hazard of nonoperative management is secondary fragment dislocation and malunion, whereas surgical treatment may result in surgery-related complications such as infection or implant failure.^21^ These options should be discussed with patients; however, there is sparse evidence on optimal management since only case reports and small case series are published to date.^18^^,^^39^^,^^58^^,^^60^ Therefore, the options of operative versus nonoperative management remain subject of ongoing debate.^35^^,^^37^^,^^54^^,^^58^ Within this paucity of literature, there seems to be consensus that LT fractures displaced more than 1 centimeter should be treated surgically.^65^ However, some studies suggest that surgeons should opt for surgical treatment if the amount of displacement is more than 5 mm, whereas other studies argue surgery for all LT fractures independent of fracture displacement due to concerns for secondary fracture displacement and impingement syndromes.^9^^,^^10^^,^^43^^,^^54^^,^^60^ While Vavken et al compared the results of arthroscopic versus open surgical treatment and demonstrated the diagnostic importance of physical examination and magnetic resonance imaging in skeletally immature patients, no review has been carried out to ascertain functional and radiographic outcomes after nonoperatively *versus* surgically treated pediatric nor adult patients with an LT fracture.^82^

Therefore, this systematic review was performed to address the clinically relevant question: what are the patient-reported outcomes, shoulder function and complications after lesser tuberosity fractures in pediatric and adult patients, as well as patients with an associated posterior shoulder dislocation? Within these groups, identical outcomes were evaluated for nonoperative, surgical, acute and delayed treatment. It was hypothesized that there was no difference in outcomes between pediatric and adult patients, as well as patients with an associated posterior shoulder dislocation

## Materials and methods

This systematic review was written according to the PRISMA guidelines and submitted for registration in PROSPERO on January 14, 2020 (ID number 165241).^48^

### Search

A search strategy was created in collaboration with the clinical librarian (C.d.H.). Studies were identified by searching Medline/Ovid, Embase.com, Cinahl/Ebsco, the Cochrane Database of Systematic Reviews for Cochrane Central Register of Controlled Trials, SPORTDiscus/Ebsco, Web of Science, Scopus, WHO ICTRP and Clinicaltrials.gov from inception up to and including October 14, 2019. Synonyms of ‘’lesser tuberosity fracture’’, ‘’subscapularis avulsion fracture’’ were combined with corresponding index terms and adjusted for every database. Details of the search are supplied in Supplementary Appendix S I.

### Selection

Records were identified with the search specified for each database and duplicates were removed in EndNote X8 (Clarivate Analytics, Boston, MA, USA). Following this identification, 2 authors (R.S. and B.S.) independently performed the screening based on title and abstract using Rayyan—a web and mobile app for systematic reviews (Ouzzani, Doha, Qatar).^56^ Subsequently, full texts were retrieved and were assessed independently for eligibility by the same authors. After each selection phase, conflicts were resolved by discussion. If disagreement remained, the last author (M.B.) was consulted or the corresponding authors of the articles were contacted. Reference lists of the included articles were manually checked for potential additional relevant articles, and a forward reference check was performed using the Web of Science and Scopus.

### Inclusion and exclusion criteria

Randomized trials, observational studies, case reports, letters, and conference papers were eligible for this review. Articles were included if patients sustained a LT fracture of the proximal humerus which was managed nonoperatively or surgically. A LT fracture was defined as an isolated avulsed bony fragment of the lesser tuberosity independent of the size without a concomitant proximal humerus fracture.

Articles were excluded if no outcome was described, data were not extractable to answer the primary research question after contacting the corresponding authors, or if patients presented with a concomitant proximal humerus fracture such as a surgical neck or greater tuberosity fracture. Study protocols, surgical technique reports, editorials, and animal or cadaver studies were also excluded.

There were no restrictions on associated injuries (such as shoulder dislocations, biceps tendon ruptures, labral injuries or glenoid fractures), age, time to presentation, fracture displacement, type of outcome, follow-up length, language or date of publication.

### Quality assessment

The quality of case reports was assessed with the tool suggested by Murad et al and the case series were assessed with the Newcastle-Ottawa scale (NOS) for Cohort studies.^49^^,^^85^ According to Murad’s tool, case reports were evaluated on: 1) selection method, 2) ascertainment of exposure and outcome, 3) causality, and 4) reporting. The NOS entailed 1) cohort representativeness, 2) ascertainment of exposure, 3) presence of outcome at start of the study, 4) assessment of outcome, 5) follow-up length, and 6) lost to follow-up rate. The overall quality of each article was judged as poor, fair, or good and was done by 2 authors independently (R.S. and B.S.). Any discrepancies were resolved by discussion in a consensus meeting.

### Data extraction and synthesis

Data were collected in Microsoft Excel version 16.35 (Microsoft Corporation, Redmond, WA, USA). Demographic variables were extracted by the first author (R.S.), and the outcome variables were in duplicate extracted by 2 authors independently (R.S. and B.S.). Variables extracted in duplicate were follow-up length, pain, satisfaction, patient-reported outcomes measures (PROMs), range of motion (ROM), strength, complications, radiological assessment and return to sport, work and daily life activities. If individual patient data was not extractable but required to answer the research questions the corresponding authors were contacted. If the value of fracture displacement was not reported within an article, computed tomography (CT) or magnetic resonance imaging images presented in the article were appreciated under supervision of a senior author (M.B. and J.D.). If CT or magnetic resonance imaging images were not provided this value was reported as missing. PROMs were combined and expressed as a percentage of 100. The variables pain, strength, range of motion, and radiographic assessment were categorized into binary variables. For instance, if a patient reported any pain at follow-up this was reported as ‘’pain’’ and if a patient reported any muscle weakness at follow-up this was reported as ‘’no full strength’’. Radiographic outcomes were categorized into union or nonunion and outcomes reporting on ROM were categorized into restricted or nonrestricted movement according to the cutoff values for elevation, abduction, and internal rotation provided in the Constant Murley Score.^12^ External rotation was categorized according to the Rowe score.^67^

### Statistical analysis

Statistical analysis was performed using IBM SPSS software version 25 (IBM Corp., Armonk, NY, USA). Categorical variables were presented as numbers with percentages, and continuous variables as means with standard deviation or median with range depending on the distribution. To indicate significant differences in outcomes between pediatric patients, adults and posterior shoulder dislocations, a logistic regression analysis was used for categorical dependent variables and a linear regression analysis for continuous dependent variables. Within these subgroups, outcome differences were assessed between acute compared to delayed treatment, and nonoperative compared to surgical treatment. Linear regression and logistic regression models were also used for these analyses. An additional regression analysis adjusted for country was performed to control the models for patients derived from similar cohorts. A *P* value less than 0.05 was considered to be significant.

## Results

**Figure 1:**
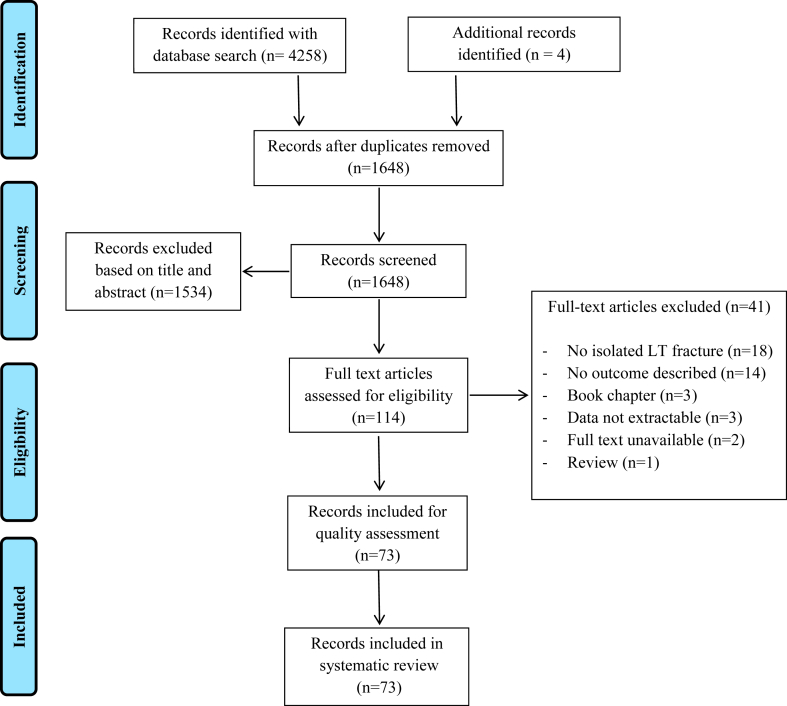
PRISMA breakdown diagram.

A total of 4258 records were identified by the database search, and 1644 records were screened for eligibility after duplicate removal. There were 110 records selected for full-text assessment and broken down to 69 records for quality assessment (Fig. 1). During full-text retrieval, 3 additional articles were found, and forward reference check revealed 164 articles of which 1 record was included.^3^^,^^30^^,^^33^^,^^68^ The overall judgment of case reports was categorized as poor in 7 articles, fair in 45 articles, and good in 9 articles (Supplementary Appendix S IIa, IIb). The quality judgment of case series ranged from fair (5 articles) to good (5 articles) (Supplementary Appendix S IIc). Given the low level of evidence of case reports and series, no articles were excluded based on the quality assessment. Taken together, 73 articles describing 71 studies were included in the systematic review^1^, ^2^, ^3^, ^4^^,^^6^^,^^7^^,^^10^^,^^11^^,^^14^^,^^15^^,^^17^, ^18^, ^19^, ^20^^,^^22^^,^^23^^,^^25^, ^26^, ^27^, ^28^, ^29^, ^30^, ^31^, ^32^, ^33^, ^34^, ^35^, ^36^, ^37^, ^38^^,^^39^, ^40^, ^41^, ^42^, ^43^, ^44^, ^45^, ^46^, ^47^^,^^50^, ^51^, ^52^, ^53^, ^54^, ^55^^,^^57^, ^58^, ^59^, ^60^, ^61^, ^62^, ^63^, ^64^^,^^66^^,^^68^, ^69^, ^70^, ^71^, ^72^, ^73^, ^74^, ^75^, ^76^, ^77^, ^78^, ^79^, ^80^, ^81^^,^^83^^,^^84^^,^^86^^,^^88^^,^^89^ (Supplementary Appendix S III).

### Cohort descriptions

Table I provides an overview of the final cohort. A total number of 175 shoulders from 172 patients were comprised in this review. There were 144 (82%) male patients of which the majority of the fractures (36%) occurred during sport. Eighty percent of patients underwent surgery and mean follow-up length was 2.1 years (range 0.08-25.0). Surgical treatment was provided to 50 of 62 (81%) pediatric patients, 49 of 66 (74%) adults, and 34 of 44 (77%) patients with an associated posterior shoulder dislocation (PSD).

**Table I:** Demographics of included patients (n = 172).

Variables	175 shoulders
Mean age at injury (range)	32.2 (9.0-77.0)
Male	144 (82.3%)
Right-sided fracture	88/153 (57.5%)
Dominant side involvement	51/80 (46.0%)
Mechanism of injury	
Sport accident	59/164 (36.0%)
Fall	29/164 (17.7%)
Seizure	25/164 (15.2%)
Traffic accident	20/164 (12.2%)
Fall from height	20/164 (12.2%)
Other∗	11/164 (6.7%)
Associated injuries	
Posterior dislocation	47 (26.9%)
BT tear or dislocation	22 (12.6%)
RC pathology	12 (6.9%)
Labrocapsular ligamentous complex injuries	9 (5.1%)
Humeral head defect	9 (5.1%)
Anterior (sub) luxation	5 (2.9%)
Other†	5 (2.9%)
Fracture displacement > 5 mm	84/101 (83.2%)
Nonoperative treatment	41 (23.4%)
Surgical treatment	134 (76.6%)
Open	119 (88.8%)
Arthroscopic	15 (11.2%)
Type of surgical fixation	
Screws	32/125 (25.6%)
Anchors	30/125 (24.0%)
Excision	22/125 (17.6%)
Modified McLaughlin	18/125 (14.4%)
Sutures	12/125 (9.6%)
Other	11/125 (8.8%)
Delayed treatment (> 6 weeks)	36/129 (27.9%)
Mean years of follow-up (range)	2.1 (0.08-25.0)

*BT*, biceps tendon; *RC*, rotator cuff.

Data are expressed as number of shoulders with percentages. If data are missing, the total number of shoulders within a variable is reported after the slash. Age was missing in 1 shoulder, length of follow-up in 6 shoulders.

∗ Assault (n = 1), no trauma reported (n = 3), syncope (n = 2), hypoglycemic fit (n = 1), electric shock (n = 4).

† Scapular spine fracture (n = 1), axillary nerve neuropraxia (n = 2), posterior glenoid rim fracture (n = 1), acromion fracture (n = 1).

### Subgroup analyses

As Table II shows, there were 62 pediatric patients, 66 adults and 44 patients with an associated PSD. In the pediatric group, 98% returned to sport, 87% regained full strength, the mean of PROMs was 94 (range 70-100) and 80% regained full ROM at follow-up. The mean of PROMs in adults was 89 (range 85-100), almost one-third (32%) had impaired range of shoulder movement in at least one plane and the complication rate was 5%. In the PSD group, 89% of shoulders did not regain full ROM and the complication rate was 17%. Unadjusted regression analysis indicated that posterior shoulder dislocations had a significantly lower mean of PROMs (*P* = .000) compared to adult patients without PSD. When stratified for country the regression analysis indicated no significant association between patients with a PSD and PROMs compared to adults (*P* = .10). Results of the sensitivity analysis are supplied in Supplementary Appendix S IV.

**Table II:** Outcomes of the pediatric (n = 62), adult (n = 66), and PSD group (n = 44).

Variable	Paediatric	Adults		PSD	
	62 shoulders	66 shoulders	*P* value	47 shoulders	*P* value
Mean age at injury (range)	13.0 (9.0-17.0)	41.3 (18.0-71.0)	**.000**	44.8 (28.0-77.0)	**.000**
Male	58 (93.5%)	46 (69.7%)	**.002**	40 (85.1%)	.16
Right-sided fracture	33/45 (73.3%)	31/62 (50.0%)	**.017**	24/46 (52.2%)	**.039**
Number of associated injuries	19 (14 shoulders)	36 (29 shoulders)	**.042**	8 (6/9 shoulders)	**.014**
Fracture displacement > 5 mm	38/42 (90.5%)	43/54 (79.6%)	.15	3/5 (60.0%)	.08
Nonoperative treatment	12 (19.4%)	17/66 (25.8%)	.39	12/47 (25.5%)	.44
Surgical treatment	50 (80.6%)	49/66 (74.2%)	.39	35/47 (74.5%)	.44
Open	43 (86.0%)	41 (83.7%)	.75	35 (100.0%)	**.038**†
Arthroscopic	7 (14.0%)	8 (16.3%)	.75	0 (0.0%)	**.038**†
Delayed treatment (> 6 weeks)	27/55 (49.1%)	9/49 (18.4%)	**.001**	0/25 (0.0%)	**.000**†
Mean years of follow-up (range)	2.6 (0.1-25.0)	1.3 (0.1-9.5)	**.012**	2.6 (0.3-3.2)	.96
Return to sport	40/41 (97.6%)	7/7 (100.0%)	1.00†	*NR*	*n/a*
Return to work	*NR*	6/7 (85.7%)	*n/a*	2/2 (100.0%)	1.00†
Return to daily life activities	10/10 (100.0%)	9/10 (90.0%)	1.00†	4/4 (100.0%)	*n/a*
Pain	3/28 (10.7%)	7/44 (15.9%)	.54	2/6 (33.3%)	.18
Mean VAS pain‡	0.44 ± 0.7	*NR*	*n/a*	0.6 ± 0.8	.26
Restricted movement	10/50 (20.0%)	12/38 (31.6%)	.22	40/45 (88.9%)	**.000**
Full strength	27/31 (87.1%)	11/12 (91.7%)	.68	4/4 (100.0%)	1.00†
Satisfaction	8/8 (100.0%)	9/11 (81.8%)	.49†	2/2 (100.0%)	*n/a*
Mean of PROMs (range)∗	93.6 (70.0-100.0)	88.6 (85.0-100.0)	**.001**	82.3 (81.2-97.1)	**.000**
Nonunion	4/16 (25.0%)	3/13 (23.1%)	.90	0/4 (0.0%)	.54†
Complications	4 (6.5%)	3 (4.5%)	.64	8 (17.0%)	.09
Secondary surgery	2 (3.2%)	1 (1.5%)	.53	2 (4.3%)	.78

*PROMs*, patient-reported outcome measures; *PSD*, posterior shoulder dislocation; *NR*, not reported; *VAS*, visual analog scale.

Data are expressed as number of shoulders with percentages. If data are missing, the total number of shoulders within a variable is reported after the slash. The *P-*values of the unadjusted regression analysis are presented and are calculated with pediatric patients as the reference group.

Significant *P-*values are indicated in bold.

∗ PROMs of 28 shoulders were described in the pediatric group and adult group, and 24 shoulders in the PSD group.

† Data were analyzed using a Fisher's exact test.

‡ VAS pain score was described in 8 pediatric patients and 22 PSD patients.

### Outcomes of surgical compared to nonoperative treatment

The mean of PROMs in nonoperatively treated pediatric patients was 84 (70-100) and the complication rate 27%. Complications (n = 3) included only mechanical impingement syndromes due to bony exostosis of the LT of which 2 patients required surgery. The mean of PROMs in surgically treated patients was 96 (85-100) and coincided with a 2% complication rate. In addition, 96% of the cases regained full strength after surgical treatment and 25% after nonoperative treatment. Adjusted regression confirmed that full strength was significantly different (*P* = .019), favoring the surgical group (Supplementary Appendix S V). Moreover, unadjusted regression analyses revealed that surgery was associated with a significantly higher mean of PROMs (*P* = .004) and fewer complications (*P* = .021) compared to nonoperative treatment (Table III).

**Table III:** Outcomes of nonoperative and surgical treatment in pediatric patients (n = 56).

Variable	Nonoperative	Surgical	*P* value
	11 shoulders	45 shoulders	
Mean age at injury (range)	13.3 (12.0-17.0)	12.9 (9.0-17.0)	.46
Male	9 (81.8%)	43 (95.6%)	.14
Right-sided fracture	9 (81.8%)	24/34 (70.6%)	.47
Number of associated injuries	0	19 (14 shoulders)	**.049**
Fracture displacement >5 mm	4/7 (57.1%)	29/29 (100.0%)	**.005**†
Open surgical treatment	*n/a*	38 (84.4%)	*n/a*
Arthroscopic surgical treatment	*n/a*	7 (15.6%)	*n/a*
Delayed treatment (> 6 weeks)	4/11 (36.4%)	20/38 (44.4%)	.35
Mean years of follow-up (range)	7.2 (0.13-25.0)	1.81 (0.23-7.00)	**.000**
Return to sport	4/5 (80.0%)	36/36 (100.0%)	.12†
Return to daily life activities	2/2 (100.0%)	8/8 (100.0%)	*n/a*
Pain	3/9 (33.3%)	0/13 (0.0%)	.055†
Mean VAS pain	*NR*	0.44 ± 0.7	*n/a*
Restricted movement	4/8 (50.0%)	6/36 (16.7%)	.054
Full strength	1/4 (25.0%)	26/27 (96.3%)	**.005**
Satisfied	*NR*	8/8 (100.0%)	*n/a*
Mean of PROMs (range)	84.4 (70.0-100.0)	95.6 (85.0-100.0)	**.004**
Nonunion	3/8 (37.5%)	1/8 (12.5%)	.57†
Complications∗	3 (27.3%)	1 (2.2%)	**.021**
Secondary surgery	2 (18.2%)	0 (0.0%)	**.036**†

*PROMs*, patient-reported outcome measures; *NR*, not reported; *n/a*, not applicable.

Data are expressed as number of shoulders with percentages. If data are missing, the total number of shoulders within a variable is reported after the slash. The *P-*values of the unadjusted regression analysis are presented.

Studies by Nardo et al and Nové-Josserand et al were excluded since data on initial treatment was not extractable per case.

Follow-up length was reported in 10 shoulders in the nonoperative group and in 43 shoulders in the surgical group. PROMs of 5 shoulders were described in the nonoperative group and 23 shoulders in the surgical group. The mean VAS was reported in 5 shoulders in the surgical group.

Significant *P-*values are indicated in bold.

∗ Mechanical impingements due to bony exostosis (n = 3) was observed after nonoperative treatment. Secondary fragment displacement (n = 1) was reported after surgical treatment.

† Data were analyzed using a Fisher's exact test.

The mean of PROMs in surgically treated adults was 94 (range 85–100), 76% of the cases regained full ROM and the complication rate was 5%. In the nonoperatively treated group, 44% regained full shoulder ROM and the complication rate was 8%. Nonunion was seen in 3 patients (38%) and only observed in the nonoperative group. At follow-up, there was no statistically significant association between surgery and the outcomes as compared to nonoperative treatment among adults (Table IV).

**Table IV:** Outcomes of nonoperative and surgical treatment in adults (n = 49).

Variable	Nonoperative	Surgical	*P* value
	12 shoulders	37 shoulders	
Mean age at injury (range)	47 (18.0-68.0)	37.9 (18.0-71.0)	.06
Male	6 (50.0%)	25 (67.6%)	.28
Right-sided fracture	5 (41.7%)	15/33 (45.5%)	.82
Number of associated injuries	4 (2 shoulders)	17 (12 shoulders)	.62
Fracture displacement >5 mm	5/9 (55.6%)	32/32 (100.0%)	**.001**†
Open surgical treatment	*n/a*	34 (91.9%)	*n/a*
Arthroscopic surgical treatment	*n/a*	3 (8.1%)	*n/a*
Delayed treatment (> 6 weeks)	2 (16.7%)	7 (18.9%)	.86
Mean years of follow-up (range)	1.3 (0.2-5.0)	1.2 (0.1-9.5)	.85
Return to sport	2/2 (100.0%)	5/5 (100.0%)	*n/a*
Return to work	1/1 (100.0%)	5/6 (83.3%)	1.00†
Return to daily life activities	4/4 (100.0%)	5/6 (83.3%)	1.00†
Pain	1/8 (12.5%)	4/19 (21.1%)	.61
Restricted movement	5/9 (55.6%)	7/29 (24.1%)	.09
Full strength	4/4 (100.0%)	7/8 (87.5%)	1.00†
Satisfied	4/4 (100.0%)	5/7 (71.4%)	.49†
Mean of PROMs (range)	89.8 (85.0-95.0)	94.3 (85.0-100.0)	.20
Nonunion	3/8 (37.5%)	0/5 (0.0%)	.23†
Complications∗	1 (8.3%)	2 (5.4%)	.72
Secondary surgery	0 (0.0%)	1 (2.7%)	1.00†

*PROMs*, patient-reported outcome measures; *NR*, not reported; *n/a*, not applicable.

Data are expressed as number of shoulders with percentages. If data are missing, the total number of shoulders within a variable is reported after the slash. The *P*-values of the unadjusted regression analysis are presented.

Studies by Nardo et al and Nové-Josserand et al were excluded since data on initial treatment was not extractable per case. Follow-up length was reported in 33 shoulders in the surgical group. PROMs of 4 shoulders were described in the nonoperative group and 7 shoulders in the surgical group.

Significant *P-*values are indicated in bold.

∗ Mechanical impingement due to bony exostosis (n = 1) was observed after nonoperative treatment. Secondary fragment displacement (n=1) and frozen shoulder (n = 1) were reported after surgical treatment.

† Data were analyzed using a Fisher's exact test.

Nonoperatively and surgically treated patients with PSD had similar complication rates of 17%. In the nonoperative group, 70% had impaired shoulder movement, and in the surgical group, this percentage was 94%. Secondary surgery occurred only in the nonoperative group (n = 2; 17%). Results are shown in Table V.

**Table V:** Outcomes of nonoperative and surgical treatment in patients with a PSD (n = 44).

Variable	Nonoperative	Surgical	*P* value
	12 shoulders	35 shoulders	
Mean age at injury (range)	45.9 (29.0-77.0)	44.4 (28.0-63.0)	.67
Male	9 (75.0%)	31 (88.6%)	.27
Right-sided fracture	4 (33.3%)	20 (57.1%)	.24
Number of associated injuries	3 (3/4 shoulders)	5 (3/5 shoulders)	.67
Fracture displacement > 5 mm	0/2 (0.0%)	3/3 (100.0%)	.10†
Open surgical treatment	*n/a*	35 (100.0%)	*n/a*
Arthroscopic surgical treatment	*n/a*	0 (0.0%)	*n/a*
Delayed treatment (> 6 weeks)	0/12 (0.0%)	0/13 (0.0%)	*n/a*
Mean years of follow-up (range)	2.2 (0.82-3.0)	2.7 (0.3-3.2)	.09
Return to work	2/2 (100.0%)	*NR*	*n/a*
Return to daily life activities	2/2 (100.0%)	2/2 (100.0%)	*n/a*
Pain	1/1 (100.0%)	1/5 (20.0%)	.33†
Mean VAS pain	*NR*	0.6 ± 0.8	*n/a*
Restricted movement	7/10 (70.0%)	33/35 (94.3%)	.051
Full strength	3/3 (100.0%)	1/1 (100.0%)	*n/a*
Satisfaction	1/1 (100.0%)	1/1 (100.0%)	*n/a*
Mean of PROMs (range)	92.0 (92.0-92.0)	81.9 (81.2-97.0)	**.007**
Nonunion	0/2 (0.0%)	0/2 (0.0%)	*n/a*
Complications∗	2 (16.7%)	6 (17.1%)	.97
Secondary surgery	2 (16.7%)	0 (0.0%)	.061†

*PROMs*, patient-reported outcome measures; *NR*, not reported; *n/a*, not applicable.

Data are expressed as number of shoulders with percentages. If data are missing, the total number of shoulders within a variable is reported after the slash. The *P-*values of the unadjusted regression analysis are presented.

Studies by Nardo et al and Nové-Josserand et al were excluded since data on initial treatment was not extractable per case.

Mean of PROMs of 1 shoulder were described in the nonoperative group and 23 shoulders in the surgical group.

Significant *P-*values are indicated in bold.

∗ Closed reduction group: iatrogenic fracture (n = 1) and redislocation requiring surgery (n = 1). Surgery group: humeral head necrosis (n = 4) and dorsal suture anchor perforation (n = 1). One patient suffered an iatrogenic brachial plexus injury (n = 1) after initial reduction, before she underwent surgery.

† Data were analyzed using a Fisher's exact test.

### Outcomes of delayed compared to acute treatment

Results of pediatric and adult patients were displayed in Table VI, Table VII, Table VIII, Table IX. There was no significant difference between the outcomes of acute and delayed treatment (>6 weeks) in pediatric and adult patients, as indicated by both adjusted and unadjusted regression models (Supplementary Appendix S VI). Regression analysis showed that patients with delayed presentation had significantly more associated injuries in both the surgical (*P* = .004) and nonoperative group (*P* = .034). The most common reported injuries were biceps tendon (BT) tears, dislocations and labro-capsular ligamentous complex injuries.

**Table VI:** Outcomes of acute and delayed surgery in pediatric patients (n = 38).

Variable	Surgery		*P* value
	Acute	Delayed	
	18 shoulders	20 shoulders	
Number of associated injuries	6 (5 shoulders)	6 (4 shoulders)	.87
BT tear or dislocation	2 (11.1%)	5 (25.0%)	.36
LCLC injuries	3 (16.7%)	0 (0.0%)	.10∗
RC pathology	0.0 (0.0%)	1 (5.0%)	1.00∗
Anterior (sub) luxation	1 (5.6%)	0 (0.0%)	.47∗
Fracture displacement > 5 mm	14/14 (100.0%)	15/15 (100.0%)	*n/a*
Mean years of follow-up (range)	1.6 (0.2-6.7)	1.5 (0.4-5.0)	.79
Return to sport	16/16 (100.0%)	15/15 (100.0%)	*n/a*
Return to daily life activities	3/3 (100.0%)	3/3 (100.0%)	*n/a*
Pain	0/7 (0.0%)	0/4 (0.0%)	*n/a*
Mean VAS pain	0.2 (0.0-1.0)	0.83 (0.0- 2.0)	.26
Restricted movement	3/16 (18.8%)	3/15 (20.0%)	.93
Full strength	14/14 (100.0%)	7/8 (87.5%)	.36∗
Satisfied	5/5 (100.0%)	3/3 (100.0%)	*n/a*
Mean of PROMs (range)	95.7 (85.0-100.0)	95.6 (91.0-99.6)	.97
Nonunion	1/3 (33.3%)	0/5 (0.0%)	.38∗
Complications	0 (0.0%)	1 (5.0%)	1.00∗
Secondary surgery	0 (0.0%)	0 (0.0%)	*n/a*

*PROMs*, patient-reported outcome measures; *NR*, not reported; *n/a*, not applicable.

Data are expressed as number of shoulders with percentages. If data are missing, the total number of shoulders within a variable is reported after the slash. The *P-*values of the unadjusted regression analysis are presented.

The mean VAS score was reported in 5 acute and 3 delayed surgically treated patients. The mean of PROMs was reported in 11 acute and 7 delayed surgically treated patients, and 4 acute and 1 delayed nonoperatively treated patients. Studies by Nardo et al, Nove-josserand et al, Liu et al, Garrigues et al, Weiss et al were excluded since data on acute and delayed treatment was not extractable per case.

∗ Data were analyzed using a Fisher's exact test.

**Table VII:** Outcomes of acute and delayed nonoperative treatment in pediatric patients (n = 11).

Variable	Nonoperative		*P* value
	Acute	Delayed	
	7 shoulders	4 shoulders	
Number of associated injuries	0 (0.0%)	0 (0.0%)	*n/a*
Fracture displacement > 5 mm	3/4 (75.0%)	1/3 (33.3%)	.29
Mean years of follow-up (range)	10.3 (0.13-25.0)	13.8 (13.0-15.0)	.20
Return to sport	2/2 (100.0%)	2/3 (66.7%)	1.00∗
Return to daily life activities	2/2 (100.0%)	*NR*	*n/a*
Pain	2/6 (33.3%)	1/3 (33.3%)	1.00
Restricted movement	3/6 (50.0%)	1/2 (50.0%)	1.00
Full strength	0/2 (0.0%)	1/2 (50.0%)	1.00∗
Mean of PROMs (range)	81.8 (70.0-100.0)	95.0 (95.0-95.0)	.46
Nonunion	2/5 (40.0%)	1/3 (33.3%)	.85
Complications	2 (28.6%)	1 (25.0%)	.90
Secondary surgery	2.0 (28.6%)	0.0 (0.0%)	.49∗

*PROMs*, patient-reported outcome measures; *NR*, not reported; *n/a*, not applicable.

Data are expressed as number of shoulders with percentages. If data are missing, the total number of shoulders within a variable is reported after the slash. The *P-*values of the unadjusted regression analysis are presented. The mean of PROMs was reported in 4 acute and 1 delayed nonoperatively treated patient. Studies by Nardo et al, Nove-josserand et al, Liu et al, Garrigues et al, and Weiss et al were excluded since data on acute and delayed treatment was not extractable per case.

∗ Data were analyzed using a Fisher's exact test.

**Table VIII:** Outcomes of acute and delayed surgery in adults (n = 37).

Variable	Surgery		*P* value
	Acute	Delayed	
	30 shoulders	7 shoulders	
Number of associated injuries	9 (7 shoulders)	8 (5 shoulders)	**.004**
BT tear or dislocation	4 (13.3%)	4 (57.14%)	.34
LCLC injuries	0 (0.0%)	2 (28.6%)	**.032**∗
RC pathology	2 (6.7%)	1 (14.3%)	**.011**
Humeral head defect	1 (3.3%)	1 (14.3%)	.26
Other	2 (6.7%)	0 (0.0%)	1.00∗
Fracture displacement > 5 mm	26/26 (100.0%)	6/6 (100.0%)	*n/a*
Mean years of follow up (range)	1.3 (0.1-9.5)	0.7 (0.3-1.0)	.48
Return to sport	2/2 (100.0%)	3/3 (100.0%)	*n/a*
Return to work	5/5 (100.0%)	0/1 (0.0%)	.17∗
Return to daily life activities	5/6 (83.3%)	*NR*	*n/a*
Pain	2/14 (14.3%)	2/5 (28.6%)	.24
Restricted movement	6/23 (26.1%)	1/6 (16.7%)	.63
Full strength	7/8 (87.5%)	*NR*	*n/a*
Satisfied	2/3 (66.7%)	3/4 (75.0%)	.81
Mean of PROMs (range)	94.3 (85.0-100.0)	*NR*	*n/a*
Nonunion	0/5 (0.0%)	*NR*	*n/a*
Complications	1 (3.3%)	1 (14.3%)	.29
Secondary surgery	0.0 (0.0%)	1 (14.3%)	.19∗

*PROMs*, patient-reported outcome measures; *NR*, not reported; *n/a*, not applicable.

Data are expressed as number of shoulders with percentages. If data are missing, the total number of shoulders within a variable is reported after the slash. The *P-*values of the unadjusted regression analysis are presented.

The mean of PROMs was described in 7 acute surgically treated shoulders, 3 acute and 1 delayed nonoperatively treated shoulder.

Significant *P-*values are indicated in bold.

∗ Data were analyzed using a Fisher's exact test.

**Table IX:** Outcomes of acute and delayed nonoperative treatment in adults (n = 12).

Variable	Nonoperative		*P* value
	Acute	Delayed	
	10 shoulders	2 shoulders	
Number of associated injuries	1 (1 shoulder)	3 (1 shoulder)	**.034**
LCLC injuries	0 (0.0%)	2 (100.0%)	.17∗
Humeral head defect	0 (0.0%)	1 (50.0%)	.17∗
Other	1 (10.0%)	0 (0.0%)	1.00∗
Fracture displacement > 5 mm	4/8 (50.0%)	1/1 (100.0%)	1.00∗
Mean years of follow up (range)	0.8 (0.2-3.3)	3.5 (1.0-5.0)	**.018**
Return to sport	2/2 (100.0%)	*NR*	*n/a*
Return to work	1/1 (100.0%)	*NR*	*n/a*
Return to daily life activities	4/4 (100.0%)	*NR*	*n/a*
Pain	0/7 (0.0%)	1/1 (100.0%)	.13∗
Restricted movement	4/8 (50.0%)	0/1 (0.0%)	1.00∗
Full strength	4/4 (100.0%)	*NR*	*n/a*
Satisfied	4/4 (100.0%)	*NR*	*n/a*
Mean of PROMs (range)	88.0 (85.0-94.0)	95.0 (95.0-95.0)	.36
Nonunion	2/5 (71.4%)	1/1 (100.0%)	.38∗
Complications	0 (0.0%)	1 (50.0%)	.17∗
Secondary surgery	0.0 (0.0%)	0.0 (0.0%)	*n/a*

*PROMs*, patient-reported outcome measures; *NR*, not reported; *n/a*, not applicable.

Data are expressed as number of shoulders with percentages. If data are missing, the total number of shoulders within a variable is reported after the slash. The *P-*values of the unadjusted regression analysis are presented.

The mean of PROMs was described in 3 acute and 1 delayed nonoperatively treated shoulder.

Significant *P-*values are indicated in bold.

∗ Data were analyzed using a Fisher's exact test.

## Discussion

LT fractures are relatively rare, with an incidence of 0.46 per 100,000 persons per year. Moreover, options of operative *versus* nonoperative management of minimally displaced LT fractures remain subject of ongoing debate.^65^ To the best of our knowledge, this study identified all reported patients and adds to literature since existing studies have drawn different conclusions on this issue.^16^ As illustration, some case series on adult patients report excellent surgical outcomes, whereas others observe comparable outcomes of nonoperative treatment, even in the setting of displaced fractures.^39^^,^^54^^,^^83^ In pediatric patients, the majority is treated surgically and data on outcomes of nonoperative treatment are limited. This review combines case reports and series to create a relatively large patient cohort aiming to provide an overview to compare these treatment strategies and inform patients about expected results. The objective was to answer the clinical question: what are patient-reported outcomes, shoulder function and complications after lesser tuberosity fractures in pediatric and adult patients, including patients with an associated posterior shoulder dislocation? Within these groups, identical outcomes were evaluated for nonoperative, surgical, acute and delayed treatment in order to guide surgical decision-making: should surgeons opt for surgical treatment in minimally displaced LT fractures?

Pediatric patients have excellent outcomes after LT fractures with almost all patients returned to sport, a high mean of PROMs, and a low complication rate. Similarly, this is explained by physiological benefits of children: they have a strong ability to remodel bone, and compared to adults, they have quicker fracture healing.^87^ Moreover, they respond well to surgical treatment and show significantly less complications and a higher mean of PROMs compared to nonoperative treatment. Adults have acceptable outcomes, but it should be noted that almost one-third did not regain a full ROM. There also seemed to be a trend towards a beneficial effect of surgical treatment; however, this difference was not significant with the numbers available. The complication rate of LT fractures after posterior shoulder dislocations was higher, and almost all patients had limited upper limb function at follow-up. Outcomes after delayed treated patients (> 6 weeks) were acceptable but must be interpreted with caution due to the low number of patients within this group.

Consistent with the review of Vavken et al, this study confirmed that surgical treatment of LT fractures provides excellent results in pediatric patients.^82^ Additionally, it was found that pediatric patients had better outcomes of surgical treatment compared to nonoperative treatment. For this reason, clinicians should strongly consider to treat pediatric patients surgically if LT fractures are displaced more than 5 mm.

In accordance with the well-designed case series of Robinson et al and Cottias et al, this study revealed good outcomes after surgically treated adult patients.^13^^,^^65^ Moreover, Cottias et al pointed out that almost one-third of the initial nonoperatively treated patients had to undergo surgery due to secondary fragment displacement.^13^ Therefore, these authors advocated for surgical treatment over nonoperative treatment in patients with a displaced LT fracture.^13^^,^^65^ In this review, however, surgical treatment was not associated with better outcomes compared to nonoperative treatment and unfortunately both case series were excluded because data were not extractable from patients with and without a PSD. It may be that some nonoperatively treated patients in this cohort should have been treated surgically as over half of the patients had more than 5-mm fracture displacement. This was supported by an additional analysis which showed that all adverse outcomes and events occurred in nonoperatively treated patients with more than 5 mm of displacement.

In this cohort, almost one-third of all shoulders were dislocated posteriorly, so suspicion should be raised for an LT fracture if patients present with a PSD. Viewed from a biomechanical perspective, the fracture is a result of the increased stress of the subscapularis muscle due to posterior luxation. Clinicians should also advise them about the relatively high complication rate and the likelihood that they will not regain full ROM. However, a note of caution is due here since the mean of functional outcome scores were acceptable despite patients did not regain full ROM and that outcomes were not compared between the different types of surgical treatment such as reversed shoulder prosthesis, modified McLaughlin technique or restoration of the humeral head with bone stock.^45^ It is important to bear in mind that patients with a PSD are more likely to undergo surgery due to associated reverse Hills-Sachs lesions which are associated with higher risk of recurrent PSD if left untreated.^8^

In clinical practice, clinicians should be aware of LT fractures and must assess radiographs carefully.^24^ Surgical decision-making should include fracture displacement, symptoms, and demands of the patient. The majority of data is published on surgical treatment, so clear guidelines on nonoperative treatment cannot be provided. However, to the best of our knowledge, we recommend conservative treatment for nondisplaced LT fractures and in patients not fit for surgery. If nonoperative treatment is chosen patients should be monitored closely and radiographs should be taken regularly and assessed for secondary fragment displacement. If secondary displacement occurs, a low threshold for surgical treatment should be followed, in particular for adults as they have less remodeling capacity than adolescents. Arthroscopic anchor suture fixation of the facture is associated with excellent outcomes and should be performed if fragment size allows this. Alternatively, open reduction with internal screw or anchor suture or transosseous suture fixation can be performed. Cancellous bone screw fixation can be performed by judgment of the surgeon.^21^

In some cases, it can be hard to appreciate the size and degree of displacement of LT fractures. It is therefore advised to perform a CT scan when considering surgical treatment. Moreover, patients with a LT fracture may present with associated injuries such as BT dislocations or tears. For this reason, surgeons should visualize the BT during surgery and if BT pathology is suspected an ultrasound can be used in the acute clinical setting.^5^

There is an important issue for further research to determine the maximum displacement accepted for nonoperative treatment. Preferably, a multicenter, randomized controlled trial will be carried out in which patients with a minimal displaced LT fracture are allocated to either surgical or nonoperative management. However, owing to the rarity of LT fractures this is almost unfeasible. Therefore, we advise a nationwide cross-sectional study in which all hospitals document and monitor these patients for 2 years and measure outcomes with PROMs, strength, ROM and radiologic assessment. This study should also address the following questions: (1) does the shape of the fragment determines outcomes? (2) which fractures associated to PSD need surgery?

### Limitations

There are some important potential drawbacks associated with this review. First, outcome measures had to be merged due to the widespread variation of reported outcomes, so conclusions should be interpreted carefully. Second, there is limited data available since only case reports and case series are reported on this subject and, third, there is a high potential for publication bias given that LT fractures are rare and that not all patients with an LT fracture worldwide are documented and published. Fourth, regression analysis was adjusted for country as adjusting for 71 different cohorts did not fit the model. Therefore, findings for both the adjusted and unadjusted regression analysis were provided but heterogeneity of population should be taken into account (Supplementary Appendix S IV, V, VI). Finally, patients with posterior shoulder dislocations were compared to pediatric and adults patients, but should be considered as the most complex trauma group among these patients. However, within these limits, this review is a collection of the best evidence available.

## Conclusion

In clinical practice, this review can be used for patient consultation and provides an overview of expected outcomes after LT fractures. It can be concluded that pediatric patients have excellent outcomes after LT fractures and may benefit more from surgery in comparison to nonoperative treatment. While the outcomes of adults are also acceptable, it is clear that the majority of patients with a PSD have lower functional outcomes scores, impaired range of shoulder movements, and are more likely to develop complications. It also highlights the importance that good outcomes can be achieved in delayed treated patients. However, poor quality of included studies has to be taken into account.

## Disclaimers:

*Funding:* No funding was disclosed by the author(s).

*Conflicts of interest:* The authors, their immediate family, and any research foundation with which they are affiliated did not receive any financial payments or other benefits from any commercial entity related to the subject of this article.

## Supplementary data

Supplementary Appendices S I - S VI
