# Supplementary material for: What are the patient-reported outcomes, functional limitations, and complications after lesser tuberosity fractures? a systematic review of 172 patients
Source: JSES Int. 2021 Apr 20;5(4):754–64. doi: 10.1016/j.jseint.2021.02.016 (PMC8245972; doi:10.1016/j.jseint.2021.02.016)
Supplement: Supplementary Appendices S I - S VI [file mmc1.docx]

**Appendix I.** Search strategy

### Medline/Ovid, 14-10-2019

| **#** | **Query** | **Results** |
| --- | --- | --- |
| 1 | Fractures, Avulsion/ | 122 |
| 2 | "avulsion*".ab,kf,ti. | 9479 |
| 3 | 1 or 2 | 9494 |
| 4 | exp humerus/ or exp humeral fractures/ or exp shoulder fractures/ | 19191 |
| 5 | "humer*".ab,kf,ti. | 25678 |
| 6 | 4 or 5 | 31743 |
| 7 | 3 and 6 | 399 |
| 8 | ((Lesser or minor or minus) adj3 (tuberosit* or tubercle* or tuberculum)).ab,kf,ti. | 418 |
| 9 | (subscapular* and avulsion*).ab,kf,ti. | 81 |
| 10 | 7 or 8 or 9 | 804 |
| 11 | exp editorial/ | 504717 |
| 12 | 10 not 11 | 803 |

### Embase.com, 14-10-2019

| **#** | **Query** | **Results** |
| --- | --- | --- |
| #7 | (#3 OR #4 OR #5) NOT [editorial]/lim | **1061** |
| #6 | #3 OR #4 OR #5 | **1064** |
| #5 | ('subscapularis muscle'/exp OR subscapular*:ab,ti) AND avulsion*:ab,ti | **86** |
| #4 | ((lesser OR minor OR minus) NEAR/3 (tuberosit* OR tubercle* OR tuberculum)):ab,ti | **503** |
| #3 | #1 AND #2 | **571** |
| #2 | 'humerus fracture'/exp OR 'shoulder fracture'/exp OR 'humerus'/exp OR humer*:ab,ti | **39342** |
| #1 | 'avulsion injury'/exp OR avulsion:ab,ti | **12767** |

### Cinahl/Ebsco, 14-10-2019

| **#** | **Query** | **Results** |
| --- | --- | --- |
| S11 | S10 NOT PT Editorial | 235 |
| S10 | S7 OR S8 OR S9 | 236 |
| S9 | TI ( subscapular* AND avulsion* ) OR AB ( subscapular* AND avulsion* ) | 25 |
| S8 | TI ( ((lesser OR minor OR minus) N3 (tuberosit* OR tubercle* OR tuberculum)) ) OR AB ( ((lesser OR minor OR minus) N3 (tuberosit* OR tubercle* OR tuberculum)) ) | 123 |
| S7 | S3 AND S6 | 115 |
| S6 | S4 OR S5 | 7,066 |
| S5 | TI humer* OR AB humer* | 5,672 |
| S4 | (MH "Humerus") OR (MH "Humeral Fractures+") OR (MH "Shoulder Fractures+") | 4,486 |
| S3 | S1 OR S2 | 2,031 |
| S2 | TI avulsion* OR AB avulsion* | 1,977 |
| S1 | (MH "Avulsion Fractures") | 199 |

### The Cochrane Library for CENTRAL and CDSR, 14-10-2019

| **#** | **Query** | **Results** |
| --- | --- | --- |
| #1 | (avulsion* AND humer*):ti,ab,kw | 7 |
| #2 | subscapular* AND avulsion*:ti,ab,kw | 1 |
| #3 | (((lesser OR minor OR minus) NEAR/3 (tuberosit* OR tubercle* OR tuberculum))):ti,ab,kw | 21 |
| #4 | {OR #1-#3} in Cochrane Reviews | 0 |
| #5 | {OR #1-#3} in Trials | 28 |

### SPORTDiscus/Ebsco, 14-10-2019

| **#** | **Query** | **Results** |
| --- | --- | --- |
| S4 | S1 OR S2 OR S3 | 117 |
| S3 | TI ( subscapular* AND avulsion* ) OR AB ( subscapular* AND avulsion* ) | 16 |
| S2 | TI ( ((lesser OR minor OR minus) N3 (tuberosit* OR tubercle* OR tuberculum)) ) OR AB ( ((lesser OR minor OR minus) N3 (tuberosit* OR tubercle* OR tuberculum)) ) | 55 |
| S1 | (DE "AVULSION fractures" OR TI avulsion* OR AB avulsion*) AND (DE "HUMERUS" OR DE "SHOULDER injuries" OR DE "HUMERUS injuries" OR TI humer* OR AB humer*) | 61 |

### Web of Science, 14-10-2019

| **#** | **Query** | **Results** |
| --- | --- | --- |
| # 6 | (#4) NOT (#5)  *Indexes=SCI-EXPANDED, SSCI, A&HCI, ESCI Timespan=All years* | 796 |
| # 5 | (#4) *AND*DOCUMENT TYPES: (Editorial Material)  *Indexes=SCI-EXPANDED, SSCI, A&HCI, ESCI Timespan=All years* | 11 |
| # 4 | #3 OR #2 OR #1  *Indexes=SCI-EXPANDED, SSCI, A&HCI, ESCI Timespan=All years* | 807 |
| # 3 | TOPIC: (((lesser OR minor OR minus) NEAR/3 (tuberosit* OR tubercle* OR tuberculum)))  *Indexes=SCI-EXPANDED, SSCI, A&HCI, ESCI Timespan=All years* | 460 |
| # 2 | TOPIC: (subscapular* AND avulsion*)  *Indexes=SCI-EXPANDED, SSCI, A&HCI, ESCI Timespan=All years* | 78 |
| # 1 | TOPIC: (avulsion* AND humer*)  *Indexes=SCI-EXPANDED, SSCI, A&HCI, ESCI Timespan=All years* | 373 |

### Scopus, 14-10-2019

| **#** | **Query** | **Results** |
| --- | --- | --- |
| #1 | ( TITLE-ABS-KEY ( ( avulsion* AND humer* ) ) OR TITLE-ABS-KEY ( ( subscapular* AND avulsion* ) ) OR TITLE-ABS-KEY ( ( ( lesser OR minor OR minus ) W/3 ( tuberosit* OR tubercle* OR tuberculum ) ) ) ) AND ( EXCLUDE ( DOCTYPE , "ed" ) ) | 1118 |

### Clinicaltrials.gov, 14-10-2019

| **#** | **Query** | **Results** |
| --- | --- | --- |
| #1 | (tuberosity OR tubercle OR tuberculum) OR (subscapularis AND avulsion) OR (humerus AND avulsion) OR (humeral AND avulsion) | 15 |

### WHO ICTRP, 14-10-2019

| **#** | **Query** | **Results** |
| --- | --- | --- |
| #1 | tuberosit* OR tubercle* OR tuberculum | 84 |
| #2 | avulsion* AND humer* | 1 |
| #3 | #1 OR #2 | 84 |

| **Appendix IIa.**  Quality assessment of case reports using the tool suggested by Murad et al. | | | | | | | | | | | | | | |  |
| --- | --- | --- | --- | --- | --- | --- | --- | --- | --- | --- | --- | --- | --- | --- | --- |
| **Year** | **First author** | | **1** | | **2** | | **3** | | **4** | | **5** | | **Overall judgement** | |  |
| 2017 | Aagaard | | no | | yes | | yes | | no | | yes | | Fair | |  |
| 1948 | Andreasen | | yes | | yes | | yes | | no | | yes | | Good | |  |
| 2017 | Atahnasiadis | | no | | yes | | yes | | no | | yes | | Fair | |  |
| 1990 | Becker | | no | | yes | | yes | | no | | no | | Poor | |  |
| 1994 | Berbig | | no | | yes | | yes | | no | | yes | | Fair | |  |
| 2000 | Biedert | | no | | yes | | yes | | no | | yes | | Fair | |  |
| 1996 | Caniggia | | no | | yes | | yes | | no | | yes | | Fair | |  |
| 1990 | Collier | | no | | yes | | yes | | no | | no | | Poor | |  |
| 2008 | Dhawan | | no | | yes | | yes | | no | | yes | | Fair | |  |
| 1990 | Earwaker | | no | | yes | | yes | | no | | yes | | Poor | |  |
| 1998 | Fabis | | no | | yes | | yes | | no | | no | | Poor | |  |
| 2012 | Goeminne | | no | | yes | | yes | | yes | | yes | | Good | |  |
| 2015 | Gornitzky | | no | | yes | | yes | | no | | yes | | Fair | |  |
| 1944 | Haas | | no | | yes | | yes | | no | | yes | | Fair | |  |
| 2015 | Hackl | | no | | yes | | yes | | no | | yes | | Fair | |  |
| 2003 | Hayes | | no | | yes | | yes | | yes | | yes | | Good | |  |
| 2008 | Heyworth | | no | | yes | | yes | | yes | | yes | | Good | |  |
| 2012 | Hung | | no | | yes | | yes | | no | | yes | | Fair | |  |
| 2008 | Jariwala | | no | | yes | | yes | | no | | yes | | Fair | |  |
| 2019 | Jaya | | no | | yes | | yes | | no | | yes | | Fair | |  |
| 1998 | Kanso | | no | | yes | | yes | | no | | yes | | Fair | |  |
| 2012 | Kato | | no | | yes | | yes | | yes | | yes | | Good | |  |
| 2014 | Kim | | no | | yes | | yes | | no | | yes | | Fair | |  |
| 1993 | Klasson | | no | | yes | | yes | | no | | yes | | Fair | |  |
| 2007 | Kowalsky | | no | | yes | | yes | | no | | yes | | Fair | |  |
| 2006 | Kumar | | no | | yes | | no | | no | | yes | | Poor | |  |
| 1993 | Kunkel | | no | | yes | | yes | | no | | yes | | Fair | |  |
| 1993 | Kuroda | | no | | yes | | yes | | no | | yes | | Fair | |  |
| 1975 | LaBriola | | no | | yes | | yes | | no | | yes | | Fair | |  |
| 1994 | LeHuec | | no | | yes | | yes | | no | | yes | | Fair | |  |
| 1997 | Leslie | | no | | yes | | yes | | yes | | no | | Fair | |  |
| 2005 | Levine | | no | | yes | | yes | | no | | yes | | Fair | |  |
| 2017 | Liong | | no | | no | | no | | no | | no | | Poor | |  |
| 2014 | Malone | | no | | yes | | yes | | no | | yes | | Fair | |  |
| 1987 | McAuliffe | | no | | yes | | yes | | no | | yes | | Fair | |  |
| 2013 | Neogi | | no | | yes | | yes | | no | | yes | | Fair | |  |
| 2014 | Nikolaou | | no | | yes | | yes | | no | | yes | | Fair | |  |
| 2011 | Ohzono | | no | | yes | | yes | | yes | | no | | Fair | |  |
| 2008 | Pace | | no | | yes | | yes | | no | | yes | | Fair | |  |
| 1995 | Paschal | | no | | yes | | yes | | no | | yes | | Fair | |  |
| 2011 | Patrizio | | no | | yes | | yes | | no | | Yes | | Fair | |  |
| **Appendix IIb.** Quality assessment of case reports using the tool suggested by Murad et al. | | | | | | | | | | | | | | | |
| **Year** | | **First author** | | **1** | | **2** | | **3** | | **4** | | **5** | | **Overall judgement** | |
| 2013 | | Paxinos | | no | | yes | | yes | | yes | | yes | | Good | |
| 2011 | | Polousky | | no | | yes | | yes | | no | | yes | | Fair | |
| 2010 | | Provance | | no | | yes | | yes | | no | | yes | | Fair | |
| 1991 | | Recht | | no | | yes | | yes | | no | | yes | | Fair | |
| 2004 | | Reparaz | | no | | yes | | yes | | no | | yes | | Fair | |
| 1989 | | Ross | | no | | yes | | yes | | no | | yes | | Fair | |
| 2009 | | Sarraf | | no | | yes | | yes | | no | | yes | | Fair | |
| 2005 | | Scheibel | | no | | yes | | yes | | no | | yes | | Fair | |
| 2015 | | Schiltz | | no | | yes | | yes | | no | | yes | | Fair | |
| 2017 | | Sharma | | no | | yes | | yes | | no | | yes | | Fair | |
| 1984 | | Shibuya | | no | | yes | | yes | | yes | | yes | | Good | |
| 2004 | | Sikka | | no | | yes | | yes | | no | | yes | | Fair | |
| 2004 | | Sugalski | | no | | yes | | yes | | no | | yes | | Fair | |
| 2017 | | Tabrizi | | no | | yes | | yes | | no | | yes | | Fair | |
| 2012 | | Teixeira | | no | | yes | | yes | | yes | | yes | | Good | |
| 1992 | | Thieleman | | no | | yes | | yes | | no | | yes | | Fair | |
| 2011 | | Tosun | | no | | yes | | yes | | yes | | yes | | Good | |
| 1985 | | White | | no | | yes | | yes | | no | | no | | Poor | |
| 1993 | | Zanlungo | | no | | yes | | yes | | no | | yes | | Fair | |
| 2018 | | Zimmerman | | no | | yes | | yes | | no | | yes | | Fair | |
| The following domains were assessed: selection (1), ascertainment of exposure (2), ascertainment of outcome (3), length of follow-up (4) and reporting (5). If patients had less than 2 years of follow-up, this domain was judged negative. Records were judged ''good'' if there was 0 or 1 negative answer within all domains. Records were judged ''fair'' if 2 answers were negative and ''poor'' if 3 or more answers were negative. | | | | | | | | | | | | | | | |
|  | | | | | | | | | | | | | | | |

| **Appendix IIc.**  Quality assessment of case series using the Newcastle-Ottawa Scale for cohort studies. | | | | | | | | |
| --- | --- | --- | --- | --- | --- | --- | --- | --- |
| **Year** | **First author** | **1** | **2** | **3** | **4** | **5** | **6** | **Overall judgement** |
| 2013 | Garrigues | 1 | 1 | 1 | 1 | 1 | 1 | Good |
| 2014 | LaMont | 1 | 0 | 0 | 0 | 0 | 1 | Fair |
| 2015 | Liu | 1 | 1 | 1 | 0 | 1 | 1 | Good |
| 2014 | Nardo | 1 | 1 | 1 | 0 | 0 | 1 | Fair |
| 1995 | Nové-Josserand | 1 | 1 | 0 | 0 | 1 | 0 | Fair |
| 1997 | Ogawa | 1 | 1 | 1 | 0 | 1 | 1 | Good |
| 2005 | Schweighofer | 1 | 0 | 1 | 0 | 1 | 1 | Good |
| 1995 | van Laarhoven | 1 | 0 | 0 | 0 | 0 | 1 | Fair |
| 2011 | Vezeridis | 1 | 1 | 0 | 1 | 1 | 1 | Good |
| 2013 | Weiss | 1 | 0 | 0 | 0 | 0 | 1 | Fair |
| The following domains were assessed: representativeness (1), ascertainment of exposure (2), outcome present at the start of the study (3), assessment of outcome (4), follow-up length (5) and adequacy of follow-up (6). | | | | | | | | |
| Records were judged ''good'' if there were 2 or 3 stars in the selection domain and 2 or 3 stars in the outcome domain. Records were judged ''fair'' if there was 1 star in the selection domain and 1 star in the outcome domain. If there were 0 stars in the selection or outcome domain, records were judged ''poor''. | | | | | | | | |

| **Appendix IIIa.** Studies describing paediatric patients. | | | |
| --- | --- | --- | --- |
| **First Author** | **year** | **Country** | **n** |
| Aagaard | 2017 | Sweden | 1 |
| Garrigues | 2013 | USA | 5 |
| Goeminne | 2012 | Belgium | 3 |
| Gornitzky | 2015 | USA | 1 |
| Heyworth | 2008 | USA | 3 |
| Kato | 2012 | Japan | 1 |
| Klasson | 1993 | USA | 2 |
| Kunkel | 1993 | USA | 1 |
| LaMont | 2014 | USA | 5 |
| LeHuec | 1994 | France | 1 |
| Levine | 2005 | USA | 1 |
| Malone | 2014 | USA | 1 |
| Nardo | 2014 | USA | 6 |
| Neogi | 2013 | UK | 2 |
| Ogawa | 1997 | Japan | 10 |
| Paschal | 1995 | USA | 2 |
| Paxinos | 2013 | Greece | 1 |
| Polousky | 2011 | USA | 2 |
| Provance | 2010 | USA | 1 |
| Ross | 1989 | USA | 2 |
| Schiltz | 2015 | Belgium | 1 |
| Shibuya | 1984 | Japan | 1 |
| Sikka | 2004 | USA | 1 |
| Sugalski | 2004 | USA | 1 |
| Teixeira | 2012 | USA | 1 |
| Vezeridis | 2011 | USA | 8 |
| Weiss | 2013 | USA | 2 |
| White | 1985 | USA | 1 |
| Zimmerman | 2018 | Germany | 1 |

| **Appendix IIIb.**  Studies describing patients with a PSD. | | | | |
| --- | --- | --- | --- | --- |
| **First Author** | **year** | | **Country** | **n** |
| Becker | 1990 | | Germany | 1 |
| Fabis | 1998 | | Poland | 1 |
| Hayes | 2003 | | USA | 1 |
| Jariwala | 2008 | | Scotland | 1 |
| Liu | 2015 | | China | 22 |
| Patrizio | 2011 | | Italy | 1 |
| Sarraf | 2009 | | UK | 2 |
| Schweighofer | 2005 | | Germany | 16 |
| Sharma | 2017 | | India | 1 |
| Tabrizi | 2017 | | Iran | 1 |
| **Appendix IIIc.** Studies describing adult patients. | | | | |
| **First Author** | **year** | **Country** | | **n** |
| Andreasen | 1948 | India | | 1 |
| Atahnasiadis | 2017 | Greece | | 1 |
| Berbig | 1994 | Germany | | 3 |
| Biedert | 2000 | Switzerland | | 1 |
| Caniggia | 1996 | Italy | | 2 |
| Collier | 1990 | UK | | 1 |
| Dhawan | 2008 | USA | | 1 |
| Earwaker | 1990 | Australia | | 2 |
| Haas | 1944 | USA | | 1 |
| Hackl | 2015 | Germany | | 1 |
| Hung | 2012 | China | | 1 |
| Jaya | 2019 | Malaysia | | 1 |
| Kanso | 1998 | France | | 1 |
| Kim | 2014 | Korea | | 1 |
| Kowalsky | 2007 | USA | | 1 |
| Kumar | 2006 | UK | | 1 |
| Kuroda | 1993 | Japan | | 1 |
| LaBriola | 1975 | USA | | 1 |
| LaMont | 2014 | USA | | 5 |
| Leslie | 1997 | UK | | 1 |
| Liong | 2017 | Malaysia | | 1 |
| McAuliffe | 1987 | UK | | 1 |
| Nikolaou | 2014 | Greece | | 1 |
| Nové-Josserand | 1995 | France | | 17 |
| Ogawa | 1997 | Japan | | 10 |
| Ohzono | 2011 | Japan | | 1 |
| Pace | 2008 | UK | | 1 |
| Recht | 1991 | Belgium | | 1 |
| Reparaz | 2004 | Spain | | 2 |
| Scheibel | 2005 | Germany | | 1 |
| Thieleman | 1992 | Germany | | 2 |
| Tosun | 2011 | Turkey | | 2 |
| van Laarhoven | 1995 | Netherlands | | 6 |
| Zanlungo | 1993 | Italy | | 1 |

| **Appendix IV.**  Sensitivity analysis for the outcomes of the paediatric, adult and PSD group. | | | | | | | | | | | |
| --- | --- | --- | --- | --- | --- | --- | --- | --- | --- | --- | --- |
|  | | **Unadjusted regression** | | | | | **Adjusted regression** | | | | |
| **Variable** | | B | | *p*-value | | | B | | | *p*-value | |
| Pain | |  | |  | | |  | | |  | |
| Adults | | 0.46 | | 0.54 | | | 22.92 | | | 1.00 | |
| PSD | | 1.43 | | 0.18 | | | 61.95 | | | 1.00 | |
| Mean VAS pain | |  | |  | | |  | | |  | |
| PSD | | 0.16 | | 0.26 | | | 0.16 | | | 0.26 | |
| Restricted movement | |  | |  | | |  | | |  | |
| Adults | | 0.61 | | 0.22 | | | -0.49 | | | 0.60 | |
| PSD | | 3.47 | | **0.000** | | | 1.83 | | | 0.09 | |
| Full strength | |  | |  | | |  | | |  | |
| Adults | | 0.49 | | 0.68 | | | 18.09 | | | 1.00 | |
| Mean of PROMs | |  | |  | | |  | | |  | |
| Adults | | -5.00 | | **0.001** | | | -1.63 | | | 0.43 | |
| PSD | | -11.30 | | **0.000** | | | -6.89 | | | **0.034** | |
| Non-union | |  | |  | | |  | | |  | |
| Adults | | -0.11 | | 0.90 | | | 19.15 | | | 1.00 | |
| Complications | |  | |  | | |  | | |  | |
| Adults | | -0.37 | | 0.64 | | | -1.38 | | | 0.27 | |
| PSD | | 1.09 | | 0.09 | | | -1.10 | | | 0.41 | |
| Secondary surgery | |  | |  | | |  | | |  | |
| Adults | | -0.77 | | 0.53 | | | -16.89 | | | 1.00 | |
| PSD | | 0.29 | | 0.78 | | | 14.53 | | | 1.00 | |
| Results of sensitivity outcome analysis adjusted for country. Paediatric patients were used as reference group for the regression models.  Abbreviations: B, unstandardized beta coefficient; PSD, posterior shoulder dislocation; PROMs, patient reported outcome measures.       \| **Appendix Va.**  Sensitivity analysis for non-operative and surgical treatment in paediatric patients. \| \| \| \| \| \| \| \| --- \| --- \| --- \| --- \| --- \| --- \| --- \| \|  \| **Unadjusted regression** \| \| **Adjusted regression** \| \| \| \| \| **Variable** \| B \| *p*-value \| B \| \| *p*-value \| \| \| Restricted movement \| -1.61 \| 0.054 \| -1.18 \| \| 0.28 \| \| \| Full strength \| 4.36 \| **0.005** \| 3.80 \| \| **0.019** \| \| \| Mean of PROMs \| 11.22 \| **0.004** \| 3.33 \| \| 0.46 \| \| \| Complications \| -2.80 \| **0.021** \| -19.83 \| \| 1.00 \| \| \| Results of sensitivity outcome analysis adjusted for country. \| \| \| \|  \| \|  \| \| Abbreviations: B, unstandardized beta coefficient; PROMs, patient reported outcome measures. \| \| \| \| \| \| \| | | | | | | | | | | | |
|  | | | | | | | | | | | |
| **Appendix Vb.**  Sensitivity analysis for non-operative and surgical treatment in adults. | | | | | | | | | | |  |
|  | **Unadjusted regression** | | | | **Adjusted regression** | | | | | |  |
| **Variable** | B | | *p*-value | | B | | | *p*-value | | |  |
| Pain | 0.62 | | 0.61 | | 18.14 | | | 1.00 | | |  |
| Restricted movement | -1.37 | | 0.09 | | -1.53 | | | 0.33 | | |  |
| Mean of PROMs | 4.58 | | 0.20 | | 4.99 | | | 0.09 | | |  |
| Complications | -0.46 | | 0.72 | | -19.30 | | | 1.00 | | |  |
| Results of sensitivity outcome analysis adjusted for country. | | | | | |  | | |  | |  |
| Abbreviations: B, unstandardized beta coefficient; PROMs, patient reported outcome measures. | | | | | | | | | | |  |

| **Appendix Vc.**  Sensitivity analysis for non-operative and surgical treatment in patients with a PSD. | | | | | | |
| --- | --- | --- | --- | --- | --- | --- |
|  | **Unadjusted regression** | | **Adjusted regression** | | | |
| **Variable** | B | *p*-value | B | | *p*-value | |
| Restricted movement | 1.96 | 0.051 | 19.26 | | 1.00 | |
| Mean of PROMs | -10.10 | **0.007** | -10.10* | | **0.007*** | |
| Complications | 0.03 | 0.97 | -0.69 | | 0.60 | |
| Results of sensitivity outcome analysis adjusted for country. | | | |  | |  |
| *The model failed to converge when adjusting for country. Therefore, the unadjusted values are presented. Abbreviations: B, unstandardized beta coefficient; PROMs, patient reported outcome measures; PSD, posterior shoulder dislocation. | | | | | | |
|  | | | | | | |

| **Appendix VIa.** Sensitivity analysis for acute and delayed surgery in paediatric patients. | | | | | | |
| --- | --- | --- | --- | --- | --- | --- |
|  | **Unadjusted regression** | | **Adjusted regression** | | | |
| **Variable** | B | *p*-value | B | | *p*-value | |
| Mean VAS pain | 0.63 | 0.26 | 0.63* | | 0.26* | |
| Restricted movement | 0.08 | 0.93 | 0.41 | | 0.71 | |
| Mean of PROMs | -0.088 | 0.97 | 0.91 | | 0.63 | |
| Results of sensitivity outcome analysis adjusted for country. | | | |  | |  |
| * The model failed to converge when adjusting for country. Therefore, the unadjusted values are presented. | | | | | | |
| Abbreviations: B, unstandardized beta coefficient; PROMs, patient reported outcome measures. | | | | | | |

| **Appendix VIb.**  Sensitivity analysis for acute and delayed non-operative treatment in paediatric patients. | | | | | | | | | | | |
| --- | --- | --- | --- | --- | --- | --- | --- | --- | --- | --- | --- |
|  | **Unadjusted regression** | | | | | | **Adjusted regression** | | | | |
| **Variable** | B | | | *p*-value | | | B | | | *p*-value | |
| Pain | 0.00 | | | 1.00 | | | 20.50 | | | 1.00 | |
| Restricted movement | 0.00 | | | 1.00 | | | 21.20 | | | 1.00 | |
| Mean of PROMs | 13.25 | | | 0.46 | | | 10.00 | | | 0.71 | |
| Non-union | -0.29 | | | 0.85 | | | 20.50 | | | 1.00 | |
| Complications | -0.18 | | | 0.90 | | | 19.62 | | | 1.00 | |
| Results of sensitivity outcome analysis adjusted for country. Abbreviations: B, unstandardized beta  coefficient; PROMs, patient reported outcome measures. | | | | | | | | | | | |
|  | | | | | | | | | | | |
| **Appendix VIc.** Sensitivity analysis for acute and delayed surgery in adults. | | | | | | | | | | |  |
|  | **Unadjusted regression** | | | | | **Adjusted regression** | | | | |  |
| **Variable** | B | | *p*-value | | | B | | | *p*-value | |  |
| Pain | 1.39 | | 0.24 | | | -20.79 | | | 1.00 | |  |
| Restricted movement | -0.57 | | 0.63 | | | -1.55 | | | 0.33 | |  |
| Satisfaction | 0.41 | | 0.81 | | | 0.69 | | | 0.71 | |  |
| Complications | 1.58 | | 0.29 | | | 0.00 | | | 1.00 | |  |
| Results of sensitivity outcome analysis adjusted for country. Abbreviations: B, unstandardized beta. coefficient. | | | | | | | | | | |  |
|  | |  | | |  | | |  | | |  |

| **Appendix VId.**  Sensitivity analysis for acute and delayed non-operative treatment in adults. | | | | | |
| --- | --- | --- | --- | --- | --- |
|  | **Unadjusted regression** | | **Adjusted regression** | | |
| **Variable** | B | *p*-value | B | | *p*-value |
| Mean of PROMs | 7.00 | 0.36 | 7.00* | | 0.36* |
| Results of sensitivity outcome analysis adjusted for country. | | | |  | |
| * The model failed to converge when adjusting for country. Therefore, the unadjusted values are presented. Abbreviations: B, unstandardized beta coefficient; PROMs, patient reported outcome measures. | | | | | |
|  | | | | | |
